# Correlation between Parental-Reported Tooth Grinding and Sleep Disorders: Investigation in a Cohort of 741 Consecutive Children

**DOI:** 10.1155/2020/3408928

**Published:** 2020-07-30

**Authors:** M. Segù, M. Pollis, A. Santagostini, F. Meola, D. Manfredini

**Affiliations:** ^1^School of Dentistry, University of Pavia, 27100 Pavia, Italy; ^2^School of Dentistry, University of Siena, 53100 Siena, Italy; ^3^Spino D'Adda, Cremona, University of Siena, 53100 Siena, Italy

## Abstract

**Purpose:**

A possible relationship between sleep bruxism (SB) and several sleep disorders has been suggested in children, which could influence sleep quality and quality of life. This study aims to assess such correlations in a large sample of school children.

**Methods:**

Parents of 741 consecutive children aged between 8 and 12 years filled the Sleep Disturbance Scale for Children (SDSC). It evaluated 45 items grouped in 8 components: duration of night-time sleep, sleep latency, bedtime problems, sleep quality, night awakenings, nocturnal symptoms, morning symptoms, and daytime sleepiness. An item evaluating parental-reported tooth grinding was also included. Correlation analysis was performed between parental-reported tooth grinding and all the other items.

**Results:**

A significant correlation between parental-reported tooth grinding and several sleep disorders concerning bedtime problems, night awakenings, nocturnal symptoms, and morning symptoms has been found. In general, correlation strength of significant pairs was low, ranging from 0.092 (sleep apnea) to 0.234 (movement while falling asleep).

**Conclusions:**

Parental-reported tooth grinding in children is correlated, even if weakly, with some sleep disorders concerning the sphere of bedtime problems, night awakenings, nocturnal symptoms, breathing symptoms, and morning symptoms. Further studies are needed to confirm these findings, with particular regard to the consistency of correlation outcomes between the parental reports and the sleep laboratory measures.

## 1. Introduction

According to the International Expert Consensuses, bruxism is defined as a repetitive jaw-muscle activity characterized by clenching or grinding of the teeth and/or by bracing or thrusting of the mandible, and it has two distinct circadian manifestations: it can occur during sleep or during wakefulness [1, 2]. Although a large amount of the literature about sleep bruxism (SB) has been performed, only few studies focused on children. A recent systematic review reports that the prevalence of SB in children is between 3.5 and 40.6%, decreasing with age and without gender differences [3]. Also, higher prevalence rates were found in children and adolescents (3–49%) than in adults (1–15%) [4]. Despite the high variability, which could depend on the adoption of parental report strategies to evaluate tooth grinding in children and makes it hard to define an actual prevalence value, the phenomenon is surely of clinical interest.

In two systematic reviews, Guo et al. listed the many factors that have been described in association with SB in children [5, 6]. Some studies assessed the relationship between parental-reported SB and sleep disorders (e.g., bedtime problems, night awakening, nocturnal symptoms, nocturnal breathing symptoms, and morning symptoms) [7, 8], whilst some others investigated for the association with behavioral problems, such as hyperactivity, poor school performance, and attention deficit. A large investigation by Restrepo et al. suggested that some sleep disorders are associated with parental-reported tooth grinding, even if further studies are needed to refine findings [9, 10]. Amongst them, breathing disorders (e.g., snoring, lack of sleep, mouth breathing, and restless sleep), whether in association with obstructive sleep apnea syndrome or not, are raising interest [6, 11].

Based on the above premises, there is an emerging evidence of a possible relationship between SB with several issues concerning the sleep features and quality as well as the presence of comorbid conditions. This study aims to assess such correlations in a large sample of consecutive school children by means of a large questionnaire-based investigation.

## 2. Materials and Methods

This case study was conducted in a private orthodontic practice between January 2016 and May 2019. A group of 741 consecutive children (*M* = 409; *F* = 332) aged between 8 and 12 years (mean age = 11.26 ± 4.05; mean weight = 25.21 ± 11.37) underwent a clinical evaluation, including an oral and orthodontic examination, performed by the same examiner. The Sleep Disturbance Scale for Children (SDSC) was administered to the accompanying parent or relative [12]. The accompanying person filled it in the waiting room or brought it at home, if the relative did not know any information about the child's sleep conditions.

## 3. Questionnaire

The SDSC questionnaire includes 45 items grouped into eight components (duration of night-time sleep, sleep latency, bedtime problems, sleep quality, night awakenings, nocturnal symptoms, morning symptoms, and daytime sleepiness). The first two components were defined by items 1 and 2, which concern sleep duration (categorized as follows: 1 = 9–11 h; 2 = 8-9 h; 3 = 7-8 h; 4 = 5–7 h; 5 = less than 5 h) and sleep latency (categorized as follows: 1 = less than 15 min; 2 = 15–30 min; 3 = 30–45 min; 4 = 45–60 min; 5 = more than 60 min). The other items can be answered based on a five-point Likert-type ordinal scale (1 = never; 2 = occasionally (once or twice a month or less); 3 = sometimes (one or twice a week); 4 = often (three or five times a week); 5 = always (every night)). Specifically, the parental-reported tooth grinding was evaluated by the question n°33 about the presence of tooth grinding during sleep (“Does she/he grind her/his teeth during sleep?”) [12]. Selection of items was based on the clinical experience with sleep disordered children and from a review of previous sleep questionnaires reported in the literature. For more details, readers are referred to the original publication [12].

Instructions for completing the scale were always given to parents by the same investigator. The questionnaire, which takes the parents 10–15 min to complete, assesses sleep behavior and disorders that have been observed during the last 6 months of the child's life. All data have been entered in the database for statistical analysis using Delta-Dent (Outside Format) software. To evaluate the level of correlation between parental-reported tooth grinding and all the other items, the Spearman test was performed. The null hypothesis was that a correlation does not exist, and a threshold of *p* lower than 0.05 was set to reject the null hypothesis. All statistical analyses were carried out using SPSS 25.0 (IBM, Milano).

## 4. Results

There were no significant differences between boys and girls as far as age and weight were concerned (mean age: *M* = 11.3 ± 4.5 ys; *F* = 11.2 ± 3.5 ys) (mean weight: *M* = 25.9 ± 11.5 kg; *F* = 24.5 ± 11.2 kg).

Considering the subjects who answered the bruxism question (*N* = 708), 70.1% of children were not reported to have sleep-time tooth grinding, whilst in 14.4%, it was reported “occasionally,” in 7.3%, “often,” and in 4.1%, either “very often” or “always.”

The Spearman test reported a significant correlation between parental-reported tooth grinding and several sleep disorders (correlation strength in parenthesis):

 
*Bedtime problems*: movement while falling asleep (*r* = 0.234), restless leg (*r* = 0.131), falling asleep sweating (*r* = 0.133), and tingling (*r* = 0.096)
 
*Night awakenings*: waking with leg cramps (*r* = 0.120) and waking up screaming in the night (*r* = 0.155)
 
*Nocturnal symptoms:* nocturnal hyperkinesia (*r* = 0.182), unusual movements during sleep (*r* = 0.155), pains of unknown origin during sleep (*r* = 0.096), nocturnal sweating (*r* = 0.171), sleep breathing difficulties (*r* = 0.163), sleep apnea (*r* = 0.092), snoring (*r* = 0.157), nightmares (*r* = 0.152), and sleep talking (*r* = 0.231)
 
*Morning symptoms:* difficulties in waking up in the morning (*r* = 0.124) and restless sleep (*r* = 0.156)

Mean values and SD of all items considered with the corresponding *p* value and *r* value are reported in Table 1.

## 5. Discussion

As known for several years, sleep disorders are common in children [12]. According to the last International Classification of Sleep Disorders (ICSD-3), sleep-related bruxism (SB) is included among the sleep-related movement disorders [5, 13, 14].

In the attempt to implement knowledge on the topic, this study has evaluated the possible association between SB and some behaviors and conditions related to sleep in children. The study was based on parental reports as a source of information. This strategy is common to most of the literature on SB in children, as the best method to collect data in large samples. Whilst the parental report may be considered a proxy for actual SB, it must be nonetheless remarked that the use of parental questionnaires is useful to collect data on several sleep behaviors and conditions that cannot be easily measured and that the child could not be aware of (e.g., sleep terrors, sleepwalking, and sleep talking). Parental information has proven to be an effective method for the detection of behavioral and developmental problems [15], and parental reports of sleep disorders are consistent with objective measurement [16, 17].

In our study, in general terms, some significant correlations emerged, even if their strength is generally weak. For instance, a correlation between parental-reported tooth grinding and bedtime problems, such as sudden movements while falling asleep (*r* = 0.234), restless legs movements, muscular pain or tingling, and falling asleep sweating, has emerged. According to Santos-Suosa et al., SB in individuals with difficulty in sleeping may be a possible result of a nervous system excitation response accompanied by body movements [7]. Furthermore, Goncalves et al. in a cross-sectional study reported an association between SB and problems with falling asleep, with an OR of 4.1 [18]. Also, as reported above, a couple of systematic reviews supported this relation [5, 6].

In this work, night awakenings (i.e., waking with leg cramps and waking up screaming in the night) and some nocturnal symptoms, such as nocturnal hyperkinesia, unusual movements during sleep, pains of unknown origin during sleep, and sleep sweating were also mildly associated with parental-reported tooth grinding. However, it is hard to compare these findings with the literature, since these specific aspects are rarely separately from other issues that influence sleep quality. Thus, more investigations are needed on the issue of comorbid sleep movements.

Interestingly, nocturnal breathing symptoms that were under investigation in this work showed a correlation with parental-reported tooth grinding (sleep breathing difficulties, sleep apnea, and snoring). These results are consistent with literature knowledge, which suggested an association with snoring in children [7]. Guo et al. included snoring among the risk factors related to the presence of bruxism [6], as also shown by another investigation that reported a relationship with respiratory disorders during sleep [19]. One of these disorders is oral breathing syndrome, the condition in which the child breathes through the mouth, thus resulting in oropharyngeal vibration, and generating the sound of snoring. In addition, there are suggestions that possible conditions leading to nasal obstruction, such as rhinitis and sinusitis, are associated with SB [20].

Our findings reported a correlation between nightmares and parental-reported tooth grinding. Rebeiro et al. showed that nocturnal agitation and nightmares were associated with possible SB in children [21]. Furthermore, Serra-Negra and colleagues observed that 71.4% of children who woke up frightened and 75% of those who awaken in the middle of the night had possible SB [19]. Brief nightmares and awakenings during sleep with palpitations are systemic features of SB [22, 23]. Also, an association with evening chronotype could exist, but the study of chronotype and its changes as a possible source of distress requires refinement, especially as far as the phenotyping of different chronotype profiles [21].

Some authors found that somniloquy is more frequent in children with SB [8], and our findings confirm these suggestions (*r* = 0.231). A possible explanation is that talking during sleep can be linked with SB that is affected by changes in brain oxygenation, which might be impaired with mouth breathing [19]. Another explanation may be that in this scenario, mouth breathing interferes with the sleep cycle and affects cerebral oxygenation, thus leading to somniloquy and involuntary muscle contractions of the facial muscles, triggering SB [24].

As for morning symptoms, the present study showed a correlation between parental-reported tooth grinding and morning headaches and restless sleep. Again, these findings are in line with the available literature supports. Goncalves et al. found a prevalence twice greater of morning headaches in children and adolescents with SB (OR = 2.6) [18]. Also, Bortoletto et al. asserted that children with SB have a greater risk of having primary headache [25]. According to some authors, patients with SB generally have morning headaches, which may depend on repetitive muscle contractions leading to tension headache [7]. On the other hand, in a case-control study of Bruni and colleagues, nocturnal symptoms were more frequent in migraine patients, especially breathing difficulties, snoring, sleep apnea, sleep talking, bruxism, and reports of frightening dreams, while tension-type headache patients were not different from controls [26]. Restless sleep could be considered as a consequence of all sleep disorders and, indeed, as mentioned above, it is also correlated to SB in children [6, 11]. A work of Vierola and colleagues has confirmed this statement suggesting that craniofacial pain is common in prepubertal children and is more frequent in those who show restless sleep and SB [27].

Some of the limitations concerning this study should be remarked. In particular, the data are cross-sectional, thus not being suitable for any inferences on causal relationships, and the collection of information was demanded to the parents. However, in studying a large cohort, using parents as a source of clinical information is, to a certain extent, a needed strategy for exploratory purposes. Investigations getting deeper into the specific associations by means of polysomnography are a recommended upgrade for the future. Notwithstanding, questionnaire approaches cannot be completely abandoned to study sleep disturbances of school-age children for obvious reasons of feasibility and compliance. It may be used as a simple screening measure and could also be helpful in analysing the relationship between overall sleep disturbance and other variables, such as age, health status, medical diseases, psychological conditions, and cognitive performances. Within these drawbacks, it must be pointed out that the strength of correlations is actually weak (low *R* values), which suggests that significance of correlations may be actually influenced by the large sample size and deserve careful reappraisal in future studies. In addition, not having divided the sample into different subgroups related to orthodontic features may not have revealed some possible relations with certain sleep disorders. For instance, as reported by Stauffer et al., several craniofacial anatomical factors (e.g., Angle class II div. II, Angle class III, anterior open bite, posterior cross bite, macroglossia, and more) seem to predispose to some sleep breathing disorders [28]. Future studies should be focused on this topic in order to get deeper into these interesting possible correlations.

## 6. Conclusions

In conclusion, the present findings suggest that parental-reported tooth grinding in children is correlated, even if weakly, with some sleep disorders concerning the sphere of bedtime problems (e.g., movement while falling asleep, restless leg, falling asleep sweating, and tingling), night awakenings (e.g., waking with leg cramps and waking up screaming in the night), nocturnal symptoms (e.g., nocturnal hyperkinesia, unusual movements during sleep, pains of unknown origin during sleep, sleep talking, and nightmares), breathing symptoms (e.g., sleep breathing difficulties, sleep apnea, snoring, and nocturnal sweating), and morning symptoms (e.g., difficulties in waking up in the morning and restless sleep).

Further studies are needed to confirm these findings, with particular regard to the consistency of correlation outcomes between the parental reports and the sleep laboratory measures. Moreover, it might be interesting to study the differences in the same clinical sample by grouping the orthodontic features and the hereditary and anamnestic variables (i.e., presence of sleep disturbances in the parents, presence of sleep problems in infancy, etc.), or to repeat and extend this study by combining the sleep disturbance scale data with other neurobehavioral variables.

## Figures and Tables

**Table 1:** Mean values and SD of all items considered with the corresponding *p* values and *r* values.

Components	Items	*N*	Mean	SD	*p* value	*R*
Bedtime problems	Movement while falling asleep	682	1.32	0.722	0.000	0.234
	Restless leg	687	1.77	1.097	0.012	0.131
	Falling asleep sweating	679	1.73	1.137	0.000	0.133
	Tingling	686	1.16	0.577	0.001	0.096

Night awakenings	Waking up with leg cramps	687	1.08	0.405	0.000	0.120
	Waking up screaming in the night	682	1.49	0.713	0.001	0.155

Nocturnal symptoms	Nocturnal hyperkinesia	688	2.01	1.162	0.000	0.182
	Unusual movements during sleep	682	1.35	0.772	0.000	0.155
	Pains of unknown origin during sleep	685	1.07	0.306	0.015	0.096
	Nocturnal sweating	680	1.72	1.024	0.000	0.171
	Sleep breathing difficulties	687	1.41	0.847	0.002	0.163
	Sleep apnea	682	1.12	0.500	0.001	0.092
	Snoring	686	1.71	0.980	0.002	0.157
	Nightmares	690	1.20	0.498	0.000	0.152
	Sleep talking	690	1.58	0.786	0.000	0.231

Morning symptoms	Difficulties in waking up in the morning	682	2.00	1.165	0.010	0.124
	Restless sleep	683	1.63	0.852	0.000	0.156

## Data Availability

The statistical data used to support the findings of this study are available from the corresponding author upon request.
